# Adherence is an optimal factor for maximizing the effective and safe use of oral anticoagulants in patients with atrial fibrillation

**DOI:** 10.1038/s41598-022-07316-y

**Published:** 2022-03-01

**Authors:** So-Young Yang, Dong-Won Kang, Jin Hyun Nam, Eue-Keun Choi, Eui-Kyung Lee, Ju-Young Shin, Sun-Hong Kwon

**Affiliations:** 1 School of Pharmacy, Sungkyunkwan University, Suwon, Republic of Korea; 2 Department of Big Data Science, Korea University Sejong Campus, Sejong, Republic of Korea; 3 Department of Internal Medicine, Seoul National University Hospital, Seoul, Republic of Korea; 4 Department of Biohealth Regulatory Science, Sungkyunkwan University, Suwon, Republic of Korea

**Keywords:** Cardiovascular diseases, Risk factors, Outcomes research

## Abstract

Few studies assessed the association between major adverse cardiovascular events and adherence to warfarin and direct oral anticoagulants (DOACs) in patients with atrial fibrillation (AF). Therefore, we aimed to evaluate the effects of adherence to oral anticoagulants (OACs) in patients with AF using claims data (July 2014–April 2019). Using the initial 3-month medication possession rate (MPR), patients were categorized into adherent (MPR ≥ 0.8) or non-adherent (MPR < 0.8) groups. Propensity score matching of non-adherent group to adherent group was conducted for warfarin (1:1) and DOAC (1:3), respectively. Incidence of ischemic stroke, myocardial infarction (MI), intracranial hemorrhage, and all-cause death was assessed in the matched cohort (67,147 patients). The hazard ratio (HR) for adherence to OAC was estimated using the Cox proportional hazard model with adjusting covariate including age and sex. The risk for ischemic stroke, MI, and all-cause death was lower in the DOAC adherent group than in the DOAC non-adherent group (HR: 0.78; 95% confidence intervals: 0.73–0.84; 0.75, 0.60–0.94; 0.54, 0.51–0.57, respectively). Adherence to OAC was not associated with the risk of intracranial hemorrhage (1.01, 0.85–1.20). Commitment programs to improve adherence in patients with AF could maximize drug effectiveness and safety.

## Introduction

In an ever-aging era, the prevalence of atrial fibrillation (AF), characterized by irregular and rapid heart rate, is expected to grow by 2.5 times over the next 50 years^1,2^. AF is known to increase the risk of stroke and can lead to thromboembolism and heart failure^3–5^. In AF, complications such as stroke and myocardial infarction (MI) are reportedly associated with high mortality. Comorbidities such as hypertension, history of a previous stroke, and coronary heart disease are not only associated with the development of AF, but also elevate the risk of stroke^6^. According to clinical guidelines, managing the risk of fatal AF complications such as stroke and myocardial infarction is important^7^. Patients should be treated with oral anticoagulants (OACs) to prevent the occurrence of these complications. A risk-factor-based assessment should precede initiating medication treatment to manage the risk of stroke. Using the CHA2DS2-VASc clinical stroke risk score, AF patients who are eligible for OACs are identified, and those with “low stroke risk” are not recommended for antithrombotic treatment. For selecting OACs, direct oral anticoagulants (DOACs) are preferred over vitamin K antagonists, mostly warfarin, as DOACs result in a lower risk of bleeding and are less affected by time in the therapeutic range (TTR) while securing similar effectiveness when compared with warfarin^7,8^. Nevertheless, patients with AF taking anticoagulants need to be carefully observed owing to the increased risk of bleeding^9–11^.

Along with regular monitoring of warfarin use, guidelines emphasize the importance of actively promoting adherence to and persistence of DOAC treatment^7^. In previous studies, adherent use of DOACs revealed superior clinical outcomes without bleeding risk. In a retrospective observational study, the adherent DOAC users reportedly showed lower risks of ischemic stroke and systemic embolism than the non-adherent ones^12^. Conversely, the risk of stroke increased 3–4 times when patients discontinued oral anticoagulant (OAC) administration^13–15^. Increasing age, presence of comorbidities, and frequent dosing schedules have been reported as risk factors for poor adherence to OACs^16,17^. Although adherence to OACs is important in preventing stroke, studies on clinical outcomes other than stroke are limited. Besides stroke, AF can also lead to MI and even death; however, studies assessing clinical outcomes other than stroke remain limited. Considering these complications, it is crucial to evaluate the benefits of OAC adherence. Moreover, intracranial hemorrhage must be assessed to verify the safety of adherence^7^. Therefore, we aimed to evaluate the risk of ischemic stroke, MI, intracranial hemorrhage, and death following adherence to DOACs or warfarin.

## Methods

### Data source

Herein, we employed the claims data from July 1, 2014 to April 30, 2019, provided by the Health Insurance Review and Assessment Service (HIRA). The HIRA data are medical claims data covering approximately 98% of the total Korean population. The database contains information about demographic characteristics, including sex, age, insurance type, and healthcare resource utilization data, such as the type of medical procedure, diagnosis of disease, costs, and medication use. Information on medication use includes the generic name, prescription date, daily dosage, quantity, duration, and general codes^18^. Disease diagnoses were coded according to the International Classification of Disease-10th revision (ICD-10).

### Study design and population

**Figure 1:**
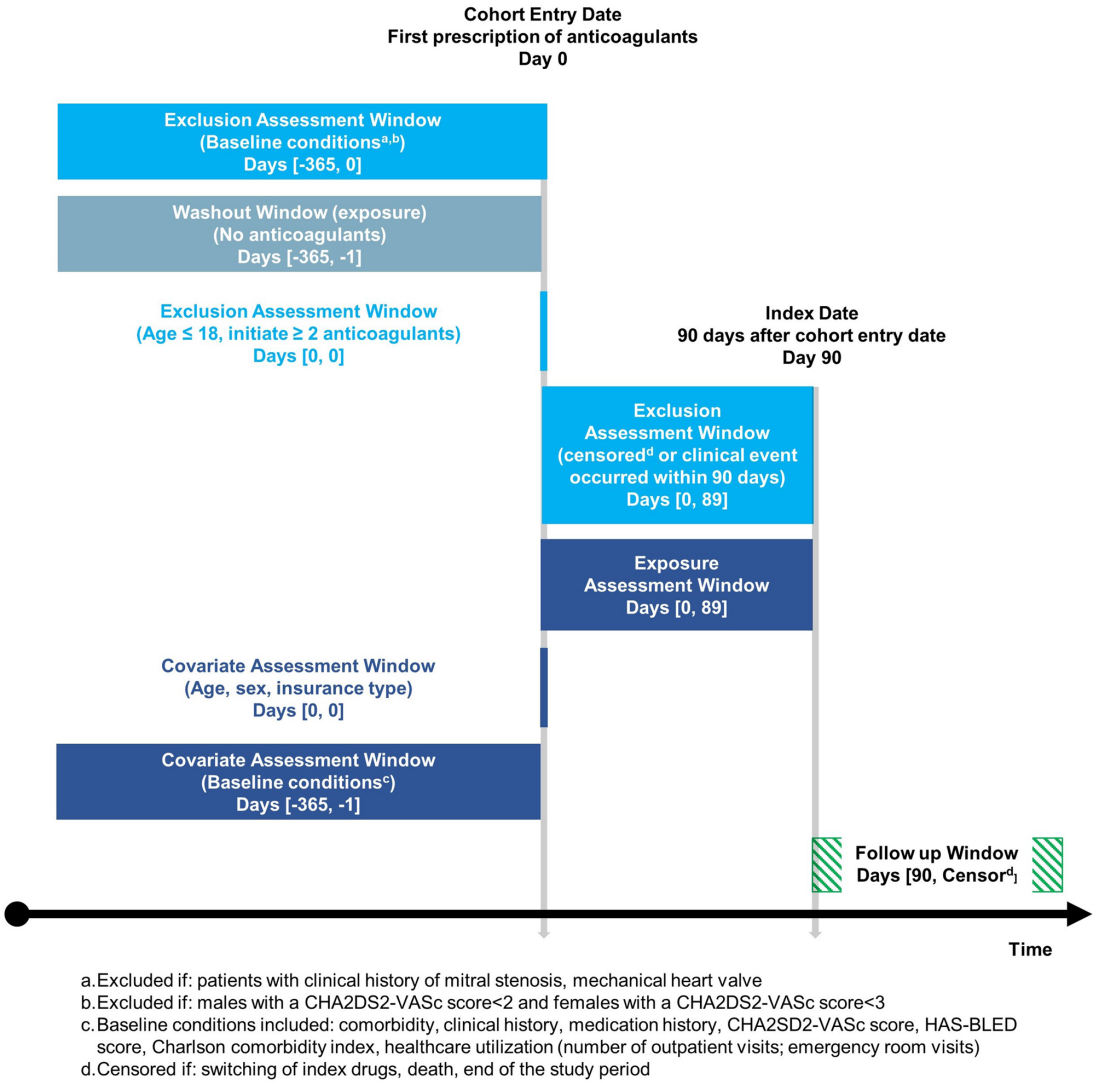
Cohort study design.

The target population in this retrospective cohort study (Fig. 1) included newly treated patients with AF who were taking OACs. To identify eligible patients from claims data, information of patients with (1) an OAC prescription and (2) a diagnosis of AF (ICD-10: I48) was extracted between July 1, 2015 and April 30, 2018^19^. OACs included warfarin and DOACs (rivaroxaban, apixaban, dabigatran, and edoxaban). The first day of OAC prescription was set as the cohort entry date. The index date was defined as 90 days after the cohort entry date. We measured adherence during this 90-day period, according to a previous study^20^. We included only those patients who did not switch drugs or did not die within 90 days of the cohort entry date.

OAC initiation is recommended based on the CHA_2_DS_2_-VAS_c_ score; this score predicts the risk of stroke and is calculated based on patient characteristics^21^. As described previously^14^, the CHA_2_DS_2_-VAS_c_ score was calculated based on the diagnosis code within a year before the cohort entry date (Supplementary Table S1). Only males with a CHA_2_DS_2_-VAS_c_ score ≥ 2 and females with a CHA_2_DS_2_-VAS_c_ score ≥ 3 were included. Only adult patients (age > 18 years) were included; patients who received OACs within 1 year before the cohort entry date were excluded, and only newly treated patients were selected. Patients diagnosed with valvular diseases (ICD-10: I05, Z952–Z954) were excluded; only patients with non-valvular AF were included^22^. Patients diagnosed with ischemic stroke, MI, and intracranial hemorrhage within 90 days of adherence determination were also excluded.

### Intervention

The medication possession rate (MPR) is used to evaluate medication adherence^23,24^ and is calculated by dividing the total number of prescription days for the initial 90 days by 90 days. Based on the MPR, the population was categorized into the adherent (MPR ≥ 0.8) or non-adherent (MPR < 0.8) groups.

As patients were categorized by early adherence, we established another cohort to determine whether early adherence to OACs continued for the later adherence. Newly treated patients with AF taking OACs were included using the same criteria as mentioned above. Among them, patients censored due to switching or death within 365 days were excluded (Supplementary Fig. S1). Pearson’s correlation test was used to assess the correlation between early and later adherence. If the Pearson correlation coefficient was close to + 1 and the *p*-value was less than 0.05, the MPR of the initial 3 months was considered to be positively correlated with the MPR in the last 3 months. The linearity was determined using a scatter plot. Odds ratios (ORs) and 95% confidence intervals (CIs) were estimated by performing univariable logistic regression analysis to assess causation between the two variables. Initial adherence was considered to affect later adherence when the *p*-value was less than 0.05.

### Outcome measurement

The patients were followed up to observe outcomes from the index date to the end of the study, death, or discontinuation of initial OACs. Outcomes were determined on the first record of the following four events independently, using ICD-10 and procedure codes: (1) ischemic stroke, (2) MI, (3) intracranial hemorrhage, and (4) death. The diagnostic codes were confirmed based on a previous study that validated the codes for clinical outcomes^25^. The operational definitions of outcomes are listed in Supplementary Table S2.

### Patient characteristics

Demographic characteristics, including sex, age group, and type of insurance on the cohort entry date, were assessed. Within 1 year before the cohort entry date, pre-clinical history of diabetes mellitus, hypertension, dyslipidemia, MI, prior percutaneous coronary intervention, prior coronary artery bypass graft, chronic renal failure, chronic obstructive pulmonary disease, unstable angina, cognitive disease, heart failure, stroke, vascular disease, intracranial hemorrhage, and cancer, which could affect the adherence to OACs or outcomes, was evaluated^17,26,27^. Concomitant medications, such as aspirin, antiplatelets, beta-blockers, and calcium channel blockers, were considered potential confounders. The operation definitions of pre-clinical history and concomitant medications are presented in Supplementary Tables S3 and S4.

The CHA_2_DS_2_-VAS_c_ and HAS-BLED scores, representative risk scores for stroke and bleeding, respectively, were calculated based on diagnosis codes within a year before the cohort entry date (Supplementary Table S1), as described previously^14^. The Charlson comorbidity index (CCI) was used to estimate the burden of underlying diseases that could affect OAC adherence^28^. Additionally, the number of emergency room (ER) visits and outpatient visits was included as a covariate for each patient.

### Statistical analysis

Propensity score (PS) matching was performed to minimize the potential impact of confounders on outcomes. Multivariable logistic regression estimated the PS for the adherent group using the following variables within a year before the cohort entry date or at the cohort entry date: age group, sex, type of insurance, pre-clinical history, co-medication, CHA_2_DS_2_-VAS_c_, HAS-BLED, CCI, and the number of ER and outpatient visits^29–33^. Matching was performed using a greedy algorithm^34^. Non-adherent warfarin users were matched 1:1 with adherent warfarin users, owing to the insufficient number of patients in the adherent group. For rivaroxaban, apixaban, dabigatran, and edoxaban, we performed 1:3 matching for each DOAC (i.e., one rivaroxaban non-adherent user was matched to three rivaroxaban adherent users). A standardized mean difference (SMD) between the adherent and non-adherent groups was estimated to compare the distribution of variables used for matching. Covariates with SMD ˃ 0.1, which can provide evidence of an imbalance between matched groups, were included in the survival analysis.

The incidence of outcomes was calculated by dividing the number of individual events by the total follow-up period and presented as 100 person-years (PY). The Cox proportional hazards model was used to estimate the adjusted hazard ratio (aHR). Age, OAC type, sex, CCI, and CHA2DS2-VASc score were included as confounders in the model. These models are presented in the Supplementary Table S6. A subgroup analysis was performed by grouping the patients as follows: (1) a group of patients aged 75 years or above, (2) a group of patients with a CHA_2_DS_2_-VAS_c_ score ≥ 4, and (3) a group of patients with CCI ≥ 4. All statistical analyses were conducted using SAS version 9.4 (SAS Institute Inc., Cary, North Carolina).

### Ethics declarations

The study was approved by the Institutional Review Board of Sungkyunkwan University (SKKU-201910031-UE001), South Korea. As patient claims data from the Health Insurance Review and Assessment Service (HIRA) had been anonymized and de-identified, the Institutional Review Board of Sungkyunkwan University waived the requirement for informed consent. The study was conducted in accordance with the Declaration of Helsinki.

### Informed consent

As patient claims data which support the findings of this study had been anonymized and de-identified publicly available information, the institutional review board waived the requirement for informed consent.

## Results

**Figure 2:**
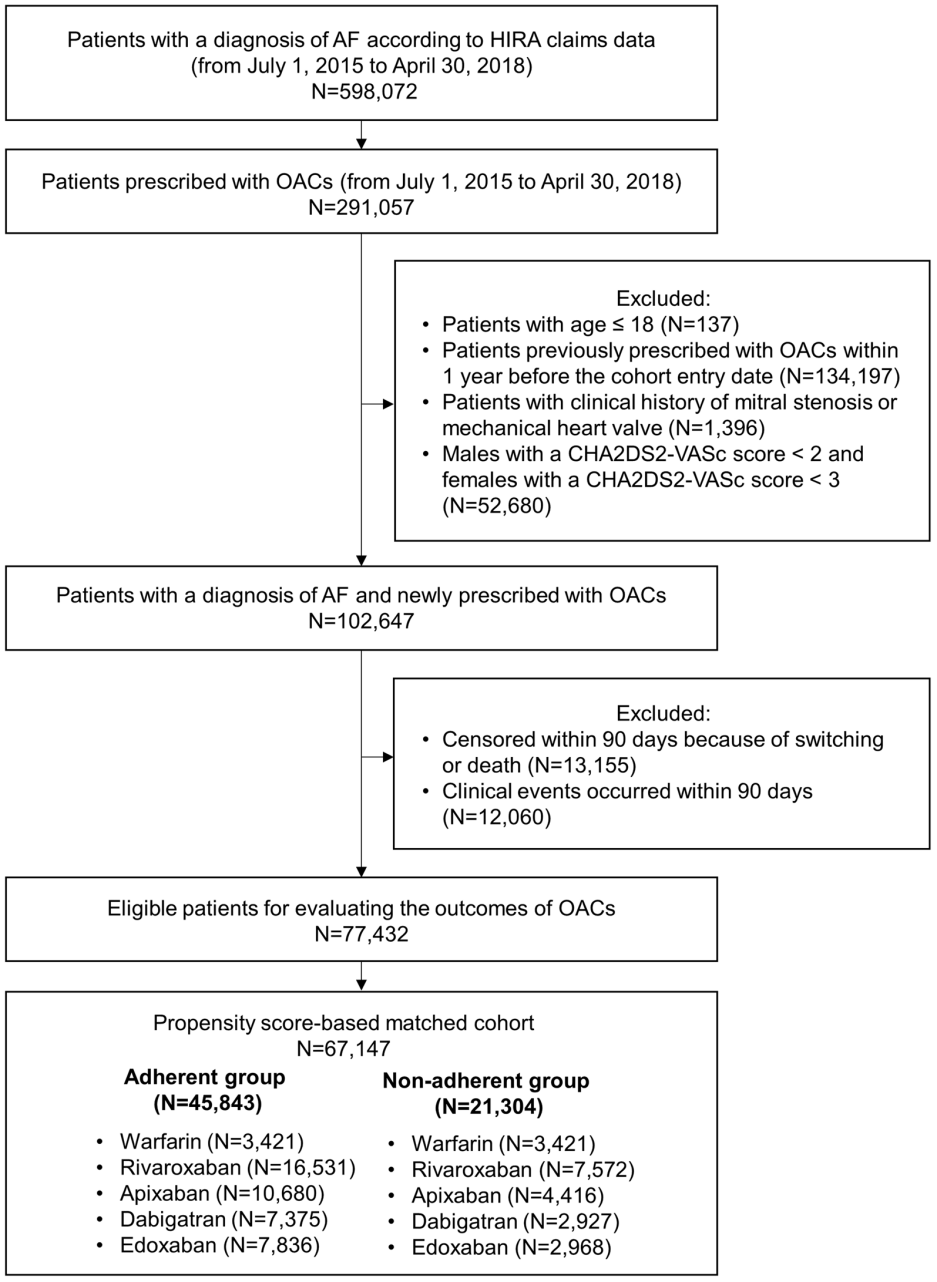
Selection of the study population. AF, atrial fibrillation; HIRA, Health Insurance Review and Assessment Service; OACs, oral anticoagulants.

In total, 67,147 patients were included after PS matching (Fig. 2). Table 1 presents the baseline characteristics of overall and matched cohorts. Additionally, patient characteristics for each drug have been shown in Supplementary Table S5. After PS matching, all SMDs, except the age group and CCI, were less than 0.1. The mean (SD) CHA2DS2-VASc score was 3.1 (0.9) in both adherent and non-adherent groups in the matched cohort.

**Table 1:** Baseline characteristics of adherent and non-adherent oral anticoagulant (OAC) users before and after propensity score matching.

	Before matching		SMD	After matching		SMD
	Adherent use (n = 55,166)	Non-adherent use (n = 22,266)		Adherent use (n = 45,843)	Non-adherent use (n = 21,304)	
**Sex, n (%)**						
Male	29,593 (53.6)	11,020 (49.5)	− 0.05	23,939 (52.2)	10,657 (50.0)	− 0.05
Female	25,573 (46.4)	11,246 (50.5)		21,904 (47.8)	10,647 (50.0)	
**Age group, n (%)**						
19–64 years	5329 (9.7)	1896 (8.5)	0.29	4204 (9.2)	1855 (8.7)	0.16
65–74 years	23,375 (42.4)	7065 (31.7)		17,224 (37.6)	6914 (32.5)	
75–84 years	22,016 (39.9)	9880 (44.4)		20,007 (43.6)	9466 (44.4)	
≥ 85 years	4446 (8.1)	3425 (15.4)		4408 (9.6)	3069 (14.4)	
**Insurance type, n (%)**						
Health insurance	50,526 (91.6)	19,762 (88.8)	0.10	41,663 (90.9)	19,075 (89.5)	0.05
Medical aid	4640 (8.4)	2504 (11.2)		4180 (9.1)	2229 (10.5)	
**Comorbidity, n (%)**						
Hypertension	42,380 (76.8)	16,993 (76.3)	0.01	35,218 (76.8)	16,402 (77.0)	0.01
Congestive heart failure	16,277 (29.5)	6416 (28.8)	0.02	13,360 (29.1)	6134 (28.8)	0.02
Diabetes mellitus	14,531 (26.3)	6078 (27.3)	− 0.02	12,357 (27.0)	5838 (27.4)	0.00
Dyslipidemia	13,112 (23.8)	4387 (19.7)	0.10	10,173 (22.2)	4294 (20.2)	0.05
Vascular disease	9317 (16.9)	4034 (18.1)	− 0.03	7908 (17.3)	3821 (17.9)	− 0.02
Ischemic stroke	8644 (15.7)	3963 (17.8)	− 0.06	7469 (16.3)	3596 (16.9)	− 0.02
Unstable angina	8737 (15.8)	2969 (13.3)	0.07	6735 (14.7)	2840 (13.3)	0.04
Cancer	4343 (7.9)	1879 (8.4)	− 0.02	3762 (8.2)	1805 (8.5)	0.00
COPD	2597 (4.7)	1276 (5.7)	− 0.05	2332 (5.1)	1190 (5.6)	− 0.02
Cognitive disease	2331 (4.2)	1349 (6.1)	− 0.08	2169 (4.7)	1230 (5.8)	− 0.04
Myocardial infarction	1607 (2.9)	601 (2.7)	0.01	1236 (2.7)	555 (2.6)	0.00
IH	382 (0.7)	211 (1)	− 0.03	350 (0.8)	192 (0.9)	− 0.02
Chronic renal failure	326 (0.6)	474 (2.1)	− 0.13	315 (0.7)	317 (1.5)	− 0.09
**Clinical history, n (%)**						
Prior PCI	696 (1.3)	287 (1.3)	0.00	545 (1.2)	261 (1.2)	− 0.01
Prior CABG	0 (0.0)	1 (0.0)	− 0.01	0 (0.0)	0 (0.0)	0.00
**Medication prescribed within 1 year before the cohort entry date, n (%)**						
Aspirin	33,137 (60.1)	11,617 (52.2)	0.16	26,184 (57.1)	11,301 (53.1)	0.08
Beta-blocker	28,702 (52.0)	9764 (43.9)	0.16	22,277 (48.6)	9467 (44.4)	0.08
CCB	25,578 (46.4)	10,702 (48.1)	− 0.03	21,528 (47.0)	10,191 (47.8)	− 0.01
Antiplatelet	21,333 (38.7)	8133 (36.5)	0.04	17,301 (37.7)	7747 (36.4)	0.03
**Risk score, mean (SD)**						
CHA_2_DS_2_-VASc	3 (0.9)	3.1 (0.9)	− 0.09	3.1 (0.9)	3.1 (0.9)	− 0.07
HAS-BLED	2 (0.7)	2.1 (0.7)	− 0.08	2 (0.7)	2.1 (0.7)	− 0.08
CCI	2.3 (1.7)	2.5 (1.9)	− 0.15	2.3 (1.8)	2.5 (1.9)	− 0.14
**Healthcare utilization within 1 year before the cohort entry date, mean (SD)**						
ER visits	0.2 (1.2)	0.3 (1.6)	− 0.05	0.2 (1.3)	0.3 (1.3)	− 0.03
Outpatient visits	35.8 (28.4)	37.4 (32.9)	− 0.05	36.1 (28.6)	37.2 (32.1)	− 0.05

SD, standard deviation; SMD, standard mean difference; PCI, percutaneous coronary intervention; CABG, coronary artery bypass grafting; COPD, chronic obstructive pulmonary disease; TIA, transient ischemic attack; TE, thromboembolism; IH, intracranial hemorrhage; CCI, Charlson comorbidity index; ER, emergency room; CCB, calcium channel blocker.

**Figure 3:**
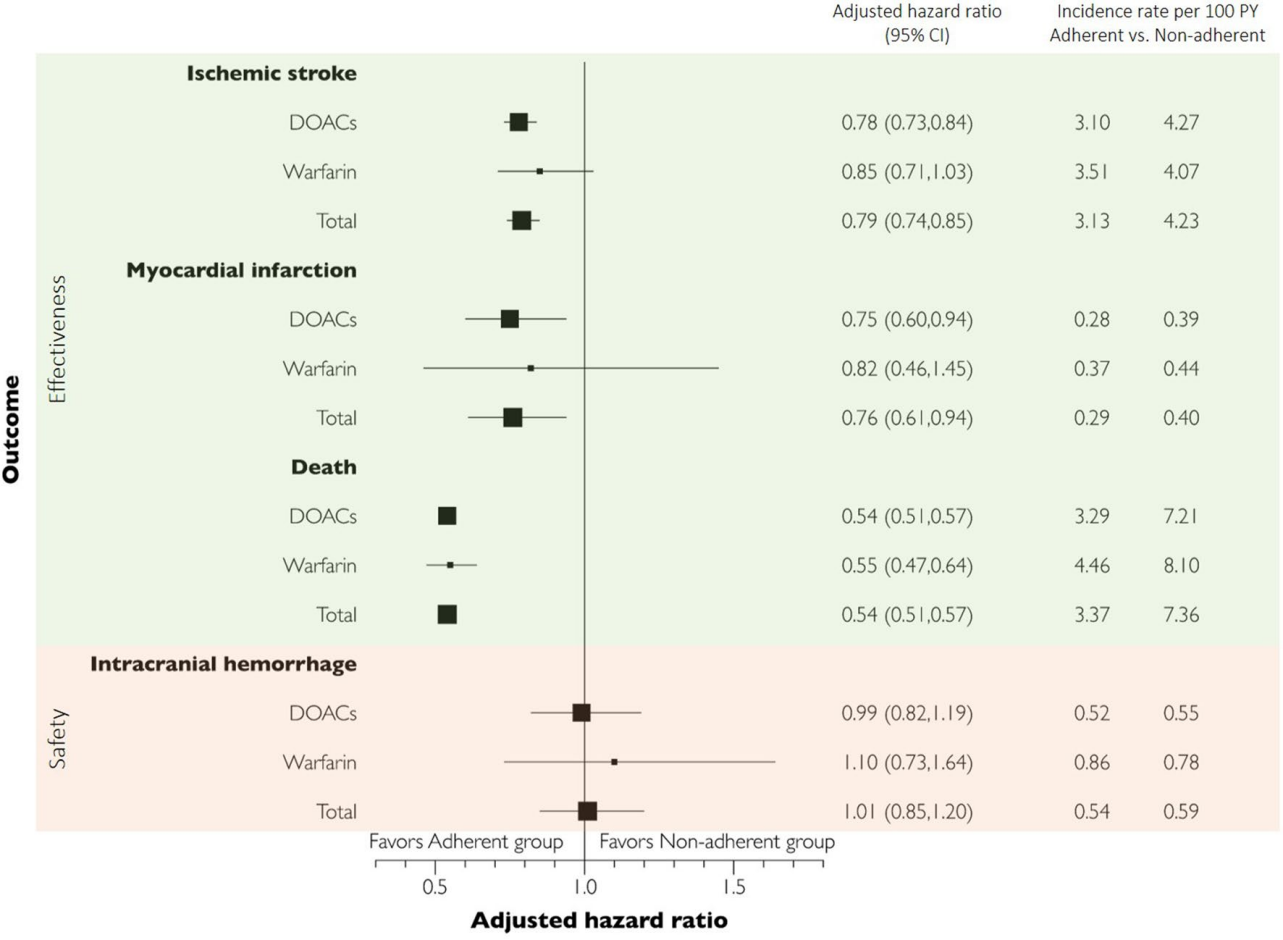
Hazard ratios of the outcomes associated with adherence to oral anticoagulants (OACs). CI, confidence interval; DOACs, direct oral anticoagulants.

The incidence rates per 100PY and aHR for events according to the adherent and non-adherent groups are shown in Fig. 3. The incidence of ischemic stroke was lower in the adherent group than in the non-adherent group (3.13 per 100PY vs. 4.23 per 100PY for DOACs or warfarin). MI and death rates were lower in the adherent group. The risk of ischemic stroke (aHR = 0.78, 95% CI 0.73–0.84), MI (0.75, 0.60–0.94), and death (0.54, 0.51–0.57) was significantly low among adherent DOAC users. Adherence to warfarin was not associated with a lower risk of ischemic stroke (0.85, 0.71–1.03) and MI (0.82, 0.46–1.45); however, a lower risk of death was noted (0.55, 0.47–0.64). The risk of intracranial hemorrhage, a known adverse outcome of OACs, was not associated with OAC adherence (1.01, 0.85–1.20). Shown in Supplementary Table S7, the aHR for ischemic stroke was not significant for the apixaban (0.87, 0.75–1.01) and dabigatran (0.85, 0.72–1.01) while it was significant for the rivaroxaban (0.76, 0.68–0.84) and edoxaban (0.66, 0.55–0.79). For MI, only edoxaban was significant (0.46, 0.24–0.86).

**Figure 4:**
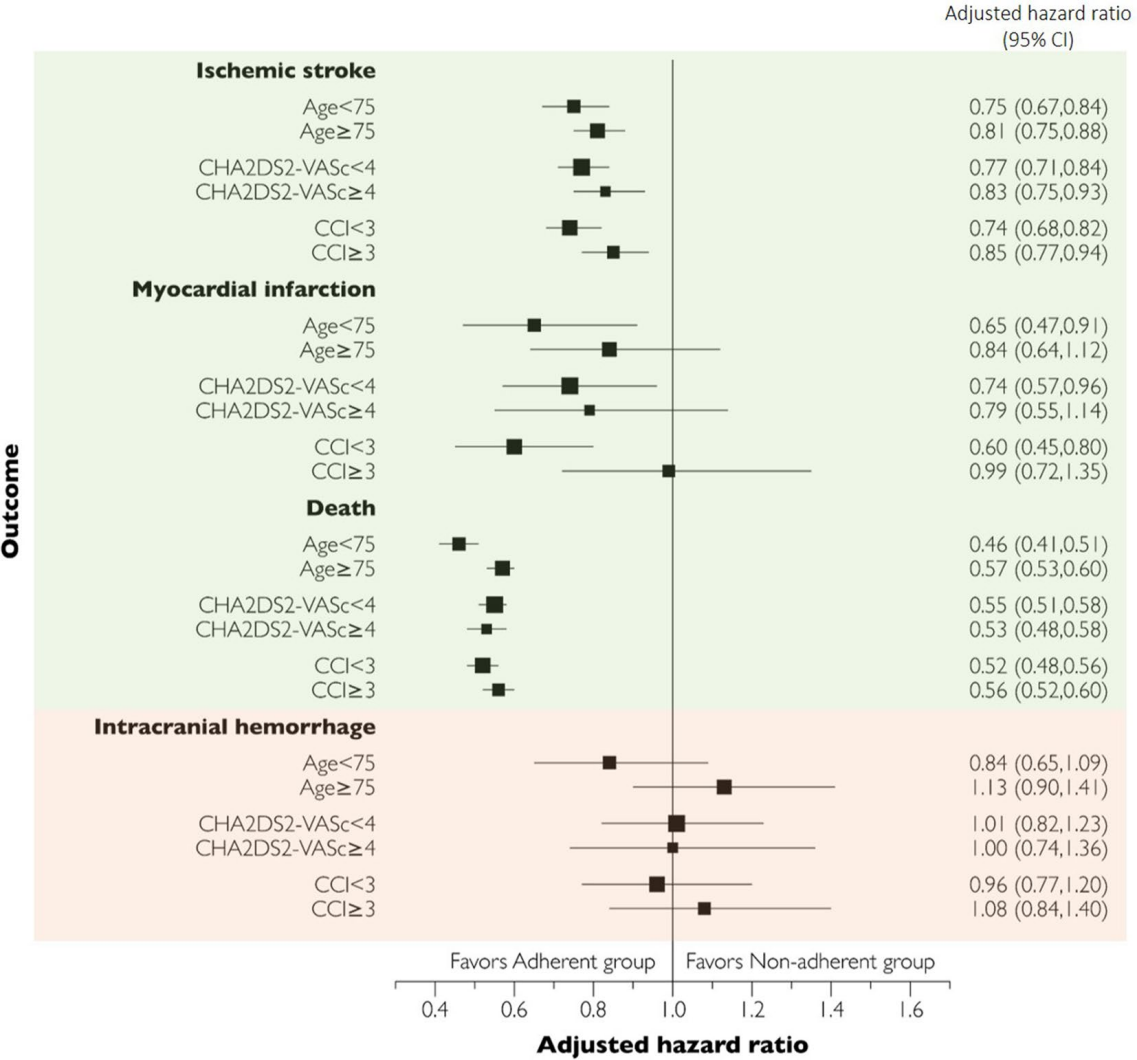
Hazard ratios of oral anticoagulants (OACs) from the subgroup analysis. CI, confidence interval.

Figure 4 shows the aHR of events in subgroups according to age group, the CHA_2_DS_2_-VAS_c_ score, and CCI. In all subgroups, the risk of ischemic stroke and death was lower in the adherent group than in the non-adherent group. The aHR for death was 0.46 (95% CI 0.41–0.51) in the younger group and 0.57 (0.53–0.60) in the elderly group. Adherence to OACs improved efficacy against MI in the lower CCI group (aHR = 0.60, 95% CI 0.45–0.80), but not in the higher CCI group (0.99, 0.72–1.35). As the type of OAC was included as a covariate in the subgroup analysis, the hazard ratio for each OAC is presented in Supplementary Table S8.

Furthermore, we assessed the association between the initial 3-month adherence and subsequent 3-month adherence (Supplementary Fig. S2). Among 76,078 patients, the Pearson correlation coefficient between the two MPR measures in the early and later periods was 0.55 (*p* < 0.0001), revealing a significantly positive correlation; this has been depicted through a scatter plot presented in Supplementary Fig. S3. Patients with high initial adherence were more likely to adhere to the prescribed treatment during the later period than patients with low initial adherence (OR 15.28; 95% CI 14.68–15.90).

## Discussion

Herein, adherence to DOACs for the initial 3 months was associated with a low risk of ischemic stroke and MI, with no increase in bleeding risk when compared with non-adherent usage. Adherence to warfarin was not associated with a reduced risk of stroke or MI, but was related to a reduced risk of death when compared with non-adherent usage.

Although the OACs themselves have suboptimal protective effects for AF patients from stroke risk, adherence was associated with better benefit of DOACs. The protective effect of adherent DOAC usage in our study was similar to that observed in previous studies. In the United States, the risk of ischemic stroke was found to be comparatively high following non-adherent DOAC usage (aHR = 1.50, 95% CI 1.30–1.73)^15^. Kim et al. also have reported results similar to those of our study. The adherent use of DOACs could be associated with the lower risk of ischemic stroke (0.73, 0.69–0.79) and MI (0.82, 0.72–0.93) without the risk of bleeding (1.01, 0.91–1.11), when compared with non-adherent use^12^. A previous retrospective study, using Medicare claims data, reported a lower HR for ischemic stroke in the adherent group (0.62) than in the non-adherent group (0.74), compared with the non-use group^14^ although it was difficult to directly compare our findings with these results. Beyond previous studies, we observed the benefit of adherence with stratifying the type of DOACs, which were on not only ischemic stroke (0.79, 0.74–0.85), MI (0.76, 0.61–0.94) but also all-cause death (0.54, 0.51–0.57).

AF complications still occurred in patients with good adherence in this study, even if the incidence rates of the adherent group were lower than those of the non-adherent group. These may also be affected by factors other than adherence, such as age or disease burden. In the subgroup analysis, the protective effect of OACs, which reduced the risk of ischemic stroke and MI, was found to be considerably high in younger patients and patients with a lower disease burden. As shown in our study, the benefit of adherent use of OACs was smaller in the older patient group than in the younger patient group when comparing point estimations. Additionally, the group with a high disease burden showed less reduction in the risk of adverse events than the low disease burden group.

Consistent with our results, Rutherford et al. have reported that the risk of stroke or bleeding is affected by age and comorbidities, as elderly patients or those with substantial comorbidities receive a reduced dose^35^. Kachroo et al. have reported that elderly patients with AF are more likely to discontinue treatment^36^. The presence of comorbidities affects not only the rate of OAC prescription, but also adherence, as previously reported that patients with high CCI are likely to demonstrate low adherence to OACs^37,38^. Previous studies also showed that stroke patients with AF who took DOACs were to be more likely to have non-modifiable factors such as being old and being female, and factors that favor thrombotic effects such as hypertension, diabetes mellitus, and dyslipidemia compared to stroke patients without AF^39,40^. Therefore, monitoring adherence in these high-risk patients is important, as recommended in clinical guidelines. Achieving optimal adherence in this patient group is essential to avoid any additional cardiovascular events, as the aforementioned patients who took DOACs were vulnerable to thrombotic factors. As reported in a recent study^41^, adding a statin to anticoagulant therapies would be worthwhile.

Interestingly, the statistical significance of aHR on adherence differed depending on the type of DOACs. Rivaroxaban and edoxaban seemed to lower the risk of ischemic stroke but apixaban and dabigatran didn’t. The difference between two types is dosing schedule. Apixaban and dabigatran are common on taking twice daily whereas rivaroxaban and edoxaban take once daily. Adherence itself can be affected by frequency of dosing^42^. This may be explored in further study.

The incidence rates of ischemic stroke attributed to DOAC adherence deviated from those previously reported. In our study, the incidence rates of ischemic stroke were 3.51 and 4.07 per 100PY in the adherent and non-adherent users, respectively, while a previous study has revealed that these rates were 1.20 and 1.92 per 100PY in the adherent and non-adherent users, respectively^14^. The deviation in incidence between studies could be attributed to patient inclusion criteria, as our study only included intermediate to high-risk patients: male patients with a CHA_2_DS_2_-VAS_c_ score ≥ 2 only and female patients with a CHA_2_DS_2_-VAS_c_ score ≥ 3 only. The study by Kim et al., which included patients with a mean CHA_2_DS_2_-VAS_c_ score of approximately 5, showed that the incidence rates of ischemic stroke were 4.21 and 5.84 per 100PY in the adherent and non-adherent groups, respectively^12^. Furthermore, racial differences may affect the incidence of stroke^43^.

Our study had several strengths. The risk of adverse health outcomes due to blood clots and the risk of bleeding, implying suboptimal control, were traced within the same dataset; therefore, the benefit of adherent OAC usage was examined from various angles. By applying the simplified study design, the findings can be interpreted easily in a cause-and-effect manner to deliver a straightforward message that adherence is related to adverse health outcomes in patients. Additionally, the results were representative of the Korean population with AF, as we used a population-level database covering the entire Korean population.

A few limitations should be considered when interpreting the present findings. First, we could not capture the data of patients who failed to administer prescribed medications despite the dispensed prescription, as adherence was estimated by prescription days using claims data. Especially for warfarin, the MPR could not serve as an alternative indicator to the period in the therapeutic range, which could be verified by the international normalized ratio (INR). However, as guidelines for patients with AF recommend that patients taking OACs should be regularly monitored by the clinician^7,44^, prescription days can be used to assess adherence to OACs, and patients with AF should be assumed to be under optimal control. In addition, as this study aimed to elucidate the clinical effect of adherence and not the therapeutic effect of OACs, factors other than adherence would have little effect on the results. Second, a misclassification bias may be present, as we used diagnostic codes of claims data to define outcomes and covariates operationally. To verify that the procedure was executed for disease remission, we limited the diagnostic codes. Furthermore, all claims of procedure codes are reviewed by HIRA to assess the appropriateness of the executing procedure before reimbursement; therefore, the procedure codes have their own accuracy and completeness. Third, the protective effect of adherence to OACs might have been overestimated, as the adherent group was older and had a slightly greater disease burden than the non-adherent group. However, the SMD revealed a balance between the adherent and non-adherent groups. Moreover, to derive the HRs of health outcomes related to adherence, we used the Cox proportional hazard model to adjust for patient characteristics, including age, sex, CCI, and the CHA_2_DS_2_-VAS_c_ score. Furthermore, we successfully verified the robustness of the study results, as the protective effect of adherent OAC use was uniform, regardless of patient characteristics, such as age group, the CHA_2_DS_2_-VAS_c_ score, and CCI, in various one-way sensitivity analyses. Comedication such as a statin may affect cardiovascular events through blood-thinning effects^41^. Although we did not observe statin use, we included dyslipidemia as one of the matching variables, and the adherent and non-adherent groups were well balanced, as shown by the SMD.

It is challenging to illustrate the impact of adherence on health outcomes because adherence is a time-varying covariate. To overcome this limitation, Hernandez et al. previously conducted a Cox proportional hazard analysis using time-dependent exposure, while Brown et al. performed the same analysis for diverse study periods, such as 3, 6, and 9 months^14,17^. In our study, we simplified the study design and simultaneously performed Pearson’s correlation test to support the assumption that adherence persisted within the first 3 months of the follow-up period. Patients with high initial adherence were shown to be more likely to adhere in the later period than those with low initial adherence. Similarly, a previous study using medical records from the outpatient clinic of a cardiology department in Korea reported that adherence to OACs was similar, regardless of the treatment period^16^. Based on the assumption of adherence persistence, we demonstrated the differences in protective effects of OACs between the adherent and non-adherent groups.

## Conclusions

Adherent use of DOACs in patients with AF could be beneficial for reducing the risk of ischemic stroke, MI, and death, without increasing the risk of bleeding. Warfarin adherence presented a lower risk of death. Adherence to OACs could be more effective in younger patients and patients with lower CCI, particularly in preventing MI. Efforts to improve adherence in patients with AF taking OACs may help reduce the burden of cardiovascular diseases.

## Supplementary Information

Supplementary Information.

## Data Availability

We used claims data from the Health Insurance Review and Assessment Service (HIRA). The claims data may be available from HIRA with permission.
