# Supplementary material for: Adherence is an optimal factor for maximizing the effective and safe use of oral anticoagulants in patients with atrial fibrillation
Source: Sci Rep. 2022 Mar 1;12:3413. doi: 10.1038/s41598-022-07316-y (PMC8888574; doi:10.1038/s41598-022-07316-y)
Supplement: Supplementary file 1 — Supplementary Information. [file 41598_2022_7316_MOESM1_ESM.docx]

**Supplementary Material**

**Article title: Adherence is an optimal factor for maximizing the effective and safe use of oral anticoagulants in patients with atrial fibrillation**

**Tables**

Table S1. Calculation of CHA2DS2-VASc and HAS-BLED scores

Table S2. Definition of outcome variables

Table S3. Definition for the pre-clinical history of the study population

Table S4. List of concomitant medications

Table S5. Baseline characteristics of adherent and non-adherent groups by oral anticoagulant subgroup after propensity score matching

Table S6. Cox proportional hazard models

Table S7. Incidence and hazard ratio by adherence

**Figures**

Figure S1. Cohort study design for identifying the correlation between early and later adherence

Figure S2. Selection of study population for identifying the association between early and later adherence.

Figure S3. Scatter plot between early and later oral anticoagulant adherence

Supplementary Table S1. Calculation of CHA_2_DS_2_-VASc and HAS-BLED scores

| Factors | Score | Operational definitions |
| --- | --- | --- |
| **CHA_2_DS_2_-VASc** |  |  |
| Congestive heart failure | 1 | One or more diagnosed records (ICD-10: I50, I110, I130, and I132) |
| Hypertension | 1 | Two or more diagnosed records (ICD-10: I10, I11, I12, I13, and I15) |
| Age ≥75 years | 2 | Age at the cohort entry date |
| Diabetes mellitus | 1 | Two requirements described below should be fulfilled:  1) Two or more diagnosed records (ICD-10: E10-E14);  2) Prescription of medication for diabetes mellitus |
| Stroke/TIA/TE | 2 | One or more diagnosed records (ICD-10: I63, I64, I66, I74, G458, and G459) |
| Valvular disease | 1 | One or more diagnosed records (ICD-10: I21, I22, I23, I70, I71, I74, I77, and I739) |
| Age 65–74 years | 1 | Age at the cohort entry date |
| Female | 1 |  |
| **HAS-BLED**† |  |  |
| Hypertension | 1 | Two or more diagnosed records (ICD-10: I10, I11, I12, I13, and I15) |
| Abnormal renal function | 1 | One or more diagnosed records (ICD-10: C64, I12, I13, N00, N01, N02, N03, N04, N05, N07, N11, N14, N17, N18, N19, N25, Q61. Z49, M103, Z940, and Z992) |
| Abnormal liver function | 1 | One or more diagnosed records (ICD-10: C22, K70-77, B150, B160, B162, B190, D684, and Z944) |
| Stroke | 1 | One or more diagnosed records (ICD-10: I63 and I64) |
| Bleeding risk | 1 | One or more diagnosed records (ICD-10: K228, K250, K252, K254, K256, K260, K262, K264, K266, K270, K272, K274, K276, K280, K282, K284, K286, K290, K625, K661, K762, K920, K921, K922, I60, I61, and I62) |
| Age ≥65 years | 1 |  |
| Abbreviations: ICD-10, International Classification of Disease 10^th^ revision; TIA, transient ischemic attack; TE, thromboembolism; INR, international normalized ratio  †The HAS-BLED is a scoring system based on (H)ypertension, (A)bnormal renal and liver function, (S)troke, (B)leeding, (L)abile INR, (E)lderly, (D)rugs or alcohol. The HAS-BLED score was calculated without evaluating INR, and alcohol consumption as the information on INR and alcohol was not included in the claims database. The drugs are presented separately in Table 1. | | |

Supplementary Table S2. Definition of outcome variables

| Covariates | Operational Definitions |
| --- | --- |
| Ischemic stroke | Two requirements described below should be fulfilled:  1) One or more diagnosed records (ICD-10: I63 and I64)  2) Presence of records with procedure code for CT, MRA, MRI (Procedure codes by HIRA: HA451, HA461, HE101, HE201, HE401, HE501, HE102, HE202, HE402, HE502, HE135, HE235, HE535, HA441, and HA471) |
| Myocardial infarction | Two requirements described below should be fulfilled:  1) One or more diagnosed records (ICD-10: I21)  2) Presence of records with procedure code for PCI, CABG, thrombolytic treatment (M6551-6554, M6561-6567, M6571 -M6572, O1640-O1642, O1647-O1649, OA640-OA642, OA647-OA649, M6634, M6638, HA670, HA680, HA681, and HA682) |
| Intracranial hemorrhage | Two requirements described below should be fulfilled:  1) One or more diagnosed records (ICD-10: I60, I61, and I62)  2) Presence of records with procedure code for CT, MRA, MRI (Procedure codes by HIRA: HA451, HA461, HE101, HE201, HE401, HE501, HE102, HE202, HE402, HE502, HE135, HE235, HE535, HA441, and HA471) |
| Death | Any of the following conditions should be met:  1) Treatment result classification code = 4 (death)  2) Diagnosis code for death (ICD-code = I461, R96, R98, and R99) |
| Abbreviations: ICD-10, International Classification of Disease 10^th^ revision; CT, computed tomography; MRA, magnetic resonance angiography; MRI, magnetic resonance imaging; HIRA, Health Insurance Review and Assessment Service; PCI, percutaneous coronary intervention; CABG, coronary artery bypass graft; | |

Supplementary Table S3. Definition for the pre-clinical history of the study population

| Covariates | Definitions |
| --- | --- |
| Diabetes mellitus | Two requirements described below should be fulfilled:  1) Two or more diagnosed records (ICD-10: E10-E14)  2) Prescription of medication for diabetes mellitus |
| Hypertension | Two or more diagnosed records (ICD-10: I10, I11, I12, I13, and I15) |
| Dyslipidemia | Two or more diagnosed records (ICD-10: E78) |
| Prior myocardial infarction | One or more diagnosed records (ICD-10: I21, I22, and I23) |
| Prior PCI | Presence of records with procedure code (M6551-M6554, M6561-M6567, M6571, and M6572) |
| Prior CABG | Presence of records with procedure code (O1640-O1642, O1647-O1649, OA640-OA642, and OA647-649) |
| Chronic renal failure | Two requirements described below should be fulfilled:  1) One or more diagnosed records (ICD-10: N18)  2) Presence of records with procedure code for dialysis (O7020, O9991, O7061, O7062, O7071, O7072, O7073, and O7074) or co-payment code for dialysis (V001 and V003) |
| COPD | Two or more diagnosed records (ICD-10: J43 and J44) |
| Unstable angina | Two or more diagnosed records (ICD-10: I20) |
| Cognitive disease | Two or more diagnosed records (ICD-10: F01, F02, F03, F04, G30, G31, R418, and G138) |
| Congestive heart failure | One or more diagnosed records (ICD-10: I50, I110, I130, and I132) |
| Ischemic stroke | One or more diagnosed records (ICD-10: I63, I64, I66, I74, G458, and G459) |
| Vascular disease | One or more diagnosed records (ICD-10: I21, I22, I23, I70, I71, I74, I77, and I739) |
| Intracranial hemorrhage | One or more diagnosed records (ICD-10: I60, I61, and I62) |
| Cancer | Two or more diagnosed records (ICD-10: C00-C99) |
| Abbreviations: ICD-10, International Classification of Disease 10^th^ revision; PCI, percutaneous coronary intervention; CABG, coronary artery bypass graft; COPD, chronic obstructive pulmonary disease  1) Cognitive diseases included Alzheimer's disease, dementia, and chronic amnesia; 2) Ischemic stroke included transient ischemic stroke and thromboembolism; 3) Vascular disease included prior myocardial infarction or peripheral artery disease. | |

Supplementary Table S4. List of concomitant medications

| Medication | Generic name | ATC code |
| --- | --- | --- |
| Aspirin | Acetylsalicylic acid | N02BA01, B01AC06 |
| Antiplatelets | Platelet aggregation inhibitors excluding heparin | B01AC |
|  | Clopidogrel | B01AC04 |
|  | Ticlopidine | B01AC05 |
|  | Indobufen | B01AC10 |
|  | Iloprost | B01AC11 |
|  | Abciximab | B01AC13 |
|  | Tirofiban | B01AC17 |
|  | Triflusal | B01AC18 |
|  | Beraprost | B01AC19 |
|  | Treprostinil | B01AC21 |
|  | Prasugrel | B01AC22 |
|  | Cilostazol | B01AC23 |
|  | Ticagrelor | B01AC24 |
|  | Selexipag | B01AC27 |
|  | Dipyridamole, acetylsalicylic acid | B01AC30 |
|  | Ticlopidine, *Ginkgo biloba* dried extract | B01AC30 |
|  | Clopidogrel, acetylsalicylic acid | B01AC30 |
| Beta-blockers | Propranolol | C07AA05 |
|  | Sotalol | C07AA07 |
|  | Nadolol | C07AA12 |
|  | Carteolol | C07AA15 |
|  | Metoprolol | C07AB02 |
|  | Atenolol | C07AB03 |
|  | Betaxolol | C07AB05 |
|  | Bevantolol | C07AB06 |
|  | Bisoprolol | C07AB07 |
|  | Celiprolol | C07AB08 |
|  | Esmolol | C07AB09 |
|  | s-Atenolol | C07AB11 |
|  | Nebivolol | C07AB12 |
|  | Alpha and beta blocking agents | C07AG |
|  | Labetalol | C07AG01 |
|  | Carvedilol | C07AG02 |
|  | Metoprolol and thiazides | C07BB02 |
|  | Bisoprolol and thiazides | C07BB07 |
|  | Atenolol and other diuretics | C07CB03 |
|  | metoprolol and felodipine | C07FB02 |
| Calcium channel blockers | Dihydropyridine derivatives | C08CA |
|  | Amlodipine | C08CA01 |
|  | Felodipine | C08CA02 |
|  | Isradipine | C08CA03 |
|  | Nicardipine | C08CA04 |
|  | Nifedipine | C08CA05 |
|  | Nimodipine | C08CA06 |
|  | Nisoldipine | C08CA07 |
|  | Lacidipine | C08CA09 |
|  | Nilvadipine | C08CA10 |
|  | Manidipine | C08CA11 |
|  | Barnidipine | C08CA12 |
|  | Lercanidipine | C08CA13 |
|  | Cilnidipine | C08CA14 |
|  | Benidipine | C08CA15 |
|  | Verapamil | C08DA01 |
|  | Diltiazem | C08DB01 |
|  | Amlodipine and diuretics | C08GA02 |
|  | Metoprolol and felodipine | C07FB02 |
|  | Metoprolol and other antihypertensives | C07FB02 |
|  | Ramipril and felodipine | C09BB05 |
|  | Losartan, amlodipine, rosuvastatin | C10BX |
|  | Telmisartan, amlodipine, rosuvastatin | C10BX |
|  | Olmesartan, amlodipine, rosuvastatin | C10BX |
|  | Amlodipine, varsartan, rosuvastatin | C10BX |
|  | Amlodipine, losartan, rosuvastatin | C10BX |
|  | Atorvastatin and amlodipine | C10BX03 |
|  | Rosuvastatin and amlodipine | C10BX09 |
| Antidiabetic drugs | Insulin (human) | A10AB01 |
|  | Insulin lispro | A10AB04 |
|  | Insulin aspart | A10AB05 |
|  | Insulin glulisine | A10AB06 |
|  | Insulin (human) | A10AC01 |
|  | Insulin lispro | A10AD04 |
|  | Insulin aspart | A10AD05 |
|  | Insulin degludec and insulin aspart | A10AD06 |
|  | Insulin glargine | A10AE04 |
|  | Insulin detemir | A10AE05 |
|  | Insulin degludec | A10AE06 |
|  | Insulin glargine and lixisenatide | A10AE54 |
|  | Metformin | A10BA02 |
|  | glibenclamide | A10BB01 |
|  | Glipizide | A10BB07 |
|  | Gliclazide | A10BB09 |
|  | Glimepiride | A10BB12 |
|  | Combinations of oral blood glucose-lowering drugs | A10BD |
|  | Metformin and sulfonylureas | A10BD02 |
|  | Glimepiride and rosiglitazone | A10BD04 |
|  | Metformin and pioglitazone | A10BD05 |
|  | Glimepiride and pioglitazone | A10BD06 |
|  | Metformin and sitagliptin | A10BD07 |
|  | Metformin and vildagliptin | A10BD08 |
|  | Pioglitazone and alogliptin | A10BD09 |
|  | Metformin and saxagliptin | A10BD10 |
|  | Metformin and linagliptin | A10BD11 |
|  | Metformin and alogliptin | A10BD13 |
|  | Metformin and repaglinide | A10BD14 |
|  | Metformin and dapagliflozin | A10BD15 |
|  | Metformin and gemigliptin | A10BD18 |
|  | Metformin and empagliflozin | A10BD20 |
|  | Acarbose | A10BF01 |
|  | Miglitol | A10BF02 |
|  | Voglibose | A10BF03 |
|  | Thiazolidinediones | A10BG |
|  | Rosiglitazone | A10BG02 |
|  | Pioglitazone | A10BG03 |
|  | Dipeptidyl peptidase 4 (DDP-4) inhibitors | A10BH |
|  | Sitagliptin | A10BH01 |
|  | Vildagliptin | A10BH02 |
|  | Saxagliptin | A10BH03 |
|  | Alogliptin | A10BH04 |
|  | Linagliptin | A10BH05 |
|  | Gemigliptin | A10BH06 |
|  | Gemigliptin and rosuvastatin | A10BH52 |
|  | Sodium-glucose co-transporter 2(SGLT2) inhibitors | A10BK |
|  | Ertugliflozin | A10BK04 |
|  | Ipragliflozin | A10BK05 |
|  | Other blood glucose-lowering drugs, excl. insulins | A10BX |
|  | Repaglinide | A10BX02 |
|  | Nateglinide | A10BX03 |
|  | Exenatide | A10BX04 |
|  | Mitiglinide | A10BX08 |
|  | Dapagliflozin | A10BX09 |
|  | Lixisenatide | A10BX10 |
|  | Empagliflozin | A10BX12 |
|  | Albiglutide | A10BX13 |
|  | Dulaglutide | A10BX14 |

Supplementary Table S5. Baseline characteristics of adherent and non-adherent groups by oral anticoagulant subgroup after propensity score matching

|  | **Adherent group (n=45,843)** | | | | | **Non-adherent group (n=21,304)** | | | | |
| --- | --- | --- | --- | --- | --- | --- | --- | --- | --- | --- |
|  | **Warfarin (n=3,421)** | **Rivaroxaban (n=16,531)** | **Apixaban (n=10,680)** | **Dabigatran (n=7,375)** | **Edoxaban (n=7,836)** | **Warfarin (n=3,421)** | **Rivaroxaban (n=7,572)** | **Apixaban (n=4,416)** | **Dabigatran (n=2,927)** | **Edoxaban (n=2,968)** |
| **Sex, n (%)** |  |  |  |  |  |  |  |  |  |  |
| Male | 1,897 (55.5) | 8,647 (52.3) | 5,245 (49.1) | 4,077 (55.3) | 4,073 (52.0) | 1,884 (55.1) | 3,625 (47.9) | 2,151 (48.7) | 1,506 (51.5) | 1,491 (50.2) |
| Female | 1,524 (44.6) | 7,884 (47.7) | 5,435 (50.9) | 3,298 (44.7) | 3,763 (48.0) | 1,537 (44.9) | 3,947 (52.1) | 2,265 (51.3) | 1,421 (48.6) | 1,477 (49.8) |
| **Age, mean (SD)** | 72.4 (9.0) | 74.3 (7.9) | 75.5 (8.1) | 73.7 (8.1) | 75.4 (8.0) | 72.6 (9.5) | 76.0 (8.4) | 77.1 (8.7) | 75.0 (9.1) | 76.5 (8.7) |
| **Age group, n (%)** |  |  |  |  |  |  |  |  |  |  |
| 19-64 years | 541 (15.8) | 1,438 (8.7) | 836 (7.8) | 781 (10.6) | 608 (7.8) | 521 (15.2) | 544 (7.2) | 286 (6.5) | 294 (10.0) | 210 (7.1) |
| 65-74 years | 1,400 (40.9) | 6,584 (39.8) | 3,566 (33.4) | 3,017 (40.9) | 2,657 (33.9) | 1,349 (39.4) | 2,445 (32.3) | 1,231 (27.9) | 990 (33.8) | 899 (30.3) |
| 75-84 years | 1,230 (36.0) | 7,044 (42.6) | 4,999 (46.8) | 3,045 (41.3) | 3,689 (47.1) | 1,284 (37.5) | 3,485 (46.0) | 2,080 (47.1) | 1,265 (43.2) | 1,352 (45.6) |
| ≥85 years | 250 (7.3) | 1,465 (8.9) | 1,279 (12.0) | 532 (7.2) | 882 (11.3) | 267 (7.8) | 1,098 (14.5) | 819 (18.6) | 378 (12.9) | 507 (17.1) |
| **Insurance type, n (%)** |  |  |  |  |  |  |  |  |  |  |
| Health insurance | 3,114 (91.0) | 15,007 (90.8) | 9,765 (91.4) | 6,675 (90.5) | 7,102 (90.6) | 3,125 (91.4) | 6,714 (88.7) | 3,958 (89.6) | 2,623 (89.6) | 2,655 (89.5) |
| Medical aid | 307 (9.0) | 1,524 (9.2) | 915 (8.6) | 700 (9.5) | 734 (9.4) | 296 (8.7) | 858 (11.3) | 458 (10.4) | 304 (10.4) | 313 (10.6) |
| **Clinical history, n (%)** | | | | | | | | | | |
| Diabetes mellitus | 938 (27.4) | 4,341 (26.3) | 3,025 (28.3) | 2,004 (27.2) | 2,049 (26.2) | 957 (28.0) | 1,998 (26.4) | 1,279 (29.0) | 824 (28.2) | 780 (26.3) |
| Hypertension | 2,567 (75.0) | 12,805 (77.5) | 8,139 (76.2) | 5,655 (76.7) | 6,052 (77.2) | 2,562 (74.9) | 5,905 (78.0) | 3,367 (76.3) | 2,281 (77.9) | 2,287 (77.1) |
| Dyslipidemia | 730 (21.3) | 3,656 (22.1) | 2,470 (23.1) | 1,656 (22.5) | 1,661 (21.2) | 732 (21.4) | 1,476 (19.5) | 931 (21.1) | 587 (20.1) | 568 (19.1) |
| Myocardial infarction | 120 (3.5) | 463 (2.8) | 322 (3.0) | 162 (2.2) | 169 (2.2) | 128 (3.7) | 176 (2.3) | 128 (2.9) | 56 (1.9) | 67 (2.3) |
| Prior PCI | 79 (2.3) | 191 (1.2) | 152 (1.4) | 57 (0.8) | 66 (0.8) | 77 (2.3) | 81 (1.1) | 59 (1.3) | 21 (0.7) | 23 (0.8) |
| Prior CABG | 0 (0) | 0 (0) | 0 (0) | 0 (0) | 0 (0) | 0 (0) | 0 (0) | 0 (0) | 0 (0) | 0 (0) |
| Chronic renal failure | 249 (7.3) | 15 (0.1) | 31 (0.3) | 7 (0.1) | 13 (0.2) | 270 (7.9) | 13 (0.2) | 23 (0.5) | 4 (0.1) | 7 (0.2) |
| COPD | 165 (4.8) | 828 (5.0) | 579 (5.4) | 382 (5.2) | 378 (4.8) | 166 (4.9) | 432 (5.7) | 256 (5.8) | 175 (6.0) | 161 (5.4) |
| Unstable angina | 506 (14.8) | 2,446 (14.8) | 1,638 (15.3) | 1,098 (14.9) | 1,047 (13.4) | 505 (14.8) | 933 (12.3) | 633 (12.3) | 404 (13.8) | 365 (12.3) |
| Cognitive disease | 125 (3.7) | 736 (4.5) | 591 (5.5) | 343 (4.7) | 374 (4.8) | 114 (3.3) | 450 (5.9) | 334 (7.6) | 167 (5.7) | 165 (5.6) |
| Congestive heart failure | 1,193 (34.9) | 4,877 (29.5) | 3,030 (28.4) | 2,051 (27.8) | 2,209 (28.2) | 1,163 (34.0) | 2,093 (27.6) | 1,239 (28.1) | 811 (27.7) | 828 (27.9) |
| Ischemic stroke | 561 (16.4) | 2,480 (15.0) | 1,913 (17.9) | 1,382 (18.7) | 1,133 (14.5) | 537 (15.7) | 1,244 (16.4) | 843 (19.1) | 540 (18.5) | 432 (14.6) |
| Vascular disease | 659 (19.3) | 2,769 (16.8) | 1,915 (17.9) | 1,210 (16.4) | 1,355 (17.3) | 683 (20.0) | 1,336 (17.6) | 772 (17.5) | 490 (16.7) | 540 (18.2) |
| IH | 24 (0.7) | 99 (0.6) | 108 (1.01) | 60 (0.81) | 59 (0.75) | 25 (0.7) | 63 (0.8) | 53 (1.2) | 26 (0.9) | 25 (0.8) |
| Cancer | 241 (7.0) | 1,297 (7.9) | 1,000 (9.4) | 556 (7.5) | 668 (8.5) | 232 (6.8) | 620 (8.2) | 478 (10.8) | 213 (7.3) | 262 (8.8) |
| **Medication prescribed within 1 year before the cohort entry date, n (%)** | | | | | | | | | | |
| Aspirin | 1,945 (56.9) | 9,893 (59.9) | 6,017 (56.3) | 4,266 (57.8) | 4,063 (51.9) | 1,941 (56.7) | 3,986 (52.6) | 2,322 (52.6) | 1,601 (54.7) | 1,451 (48.9) |
| Antiplatelet | 1,233 (36.0) | 6,135 (37.1) | 4,219 (39.5) | 2,762 (37.5) | 2,952 (37.7) | 1,252 (36.6) | 2,643 (34.9) | 1,717 (38.9) | 1,048 (35.8) | 1,087 (36.6) |
| Beta-blocker | 1,724 (50.4) | 8,096 (49.0) | 5,336 (50.0) | 3,526 (47.8) | 3,595 (45.9) | 1,725 (50.4) | 3,192 (42.2) | 2,021 (45.8) | 1,269 (43.4) | 1,260 (42.5) |
| CCB | 1,647 (48.1) | 7,436 (45.0) | 5,384 (50.4) | 3,422 (46.4) | 3,639 (46.4) | 1,655 (48.4) | 3,520 (46.5) | 2,253 (51.0) | 1,389 (47.5) | 1,374 (46.3) |
| **Risk score, mean (SD)** |  |  |  |  |  |  |  |  |  |  |
| CHA2SD2-VASc | 3 (0.9) | 3 (0.9) | 3.1 (0.9) | 3 (0.9) | 3 (0.9) | 3 (0.9) | 3.1 (0.9) | 3.2 (0.9) | 3.1 (0.9) | 3.1 (0.9) |
| HAS-BLED | 2.1 (0.8) | 2 (0.7) | 2.1 (0.7) | 2 (0.7) | 2 (0.7) | 2.1 (0.8) | 2.1 (0.7) | 2.1 (0.7) | 2 (0.7) | 2 (0.7) |
| CCI | 2.6 (1.9) | 2.3 (1.7) | 2.5 (1.8) | 2.3 (1.7) | 2.3 (1.7) | 2.7 (1.9) | 2.4 (1.8) | 2.7 (2.0) | 2.4 (1.8) | 2.4 (1.8) |
| **Healthcare utilization within 1 year before the cohort entry date, mean (SD)** | | | | | | | | | | |
| ER visits | 0.2 (1.1) | 0.2 (1.1) | 0.3 (1.9) | 0.2 (1.0) | 0.2 (1.1) | 0.2 (0.7) | 0.3 (1.0) | 0.3 (2.3) | 0.3 (0.9) | 0.3 (1.2) |
| Outpatient visits | 39.6 (36.8) | 35.9 (27.7) | 36.6 (28.1) | 35.6 (28.6) | 34.8 (26.6) | 40.8 (40.0) | 37.6 (31.0) | 36.0 (30.0) | 36.2 (30.4) | 34.6 (29.1) |
| Abbreviations: SD, standard deviation; SMD, standard mean difference; PCI, percutaneous coronary intervention; CABG, coronary artery bypass grafting; COPD, chronic obstructive pulmonary disease; TIA, transient ischemic attack; TE, thromboembolism; IH, intracranial hemorrhage; CCI, Charlson comorbidity index; ER, emergency room; CCB, calcium channel blocker | | | | | | | | | | |

**Supplementary Table S6.** Cox proportional hazard models

|  | **Hazard ratio (95% CI)** | | |
| --- | --- | --- | --- |
|  | **Warfarin** | **DOACs** | **Total** |
| **Ischemic stroke** | | | |
| Adherence (ref. non-adherence) | 0.85 (0.71, 1.03) | 0.78 (0.73, 0.84) | 0.79 (0.74, 0.85) |
| Type of OACs (ref. Warfarin) |  |  |  |
| Rivaroxaban | N/A | N/A | 0.92 (0.83, 1.03) |
| Apixaban | N/A | N/A | 0.82 (0.73, 0.93) |
| Dabigatran | N/A | N/A | 1.00 (0.88, 1.13) |
| Edoxaban | N/A | N/A | 0.81 (0.71, 0.92) |
| Sex (ref. male) | 0.93 (0.73, 1.17) | 0.81 (0.74, 0.88) | 0.83 (0.76, 0.90) |
| Age | 1.03 (1.01, 1.04) | 1.03 (1.03, 1.04) | 1.03 (1.03, 1.04) |
| CCI | 1.12 (1.07, 1.18) | 1.07 (1.05, 1.09) | 1.08 (1.06, 1.10) |
| CHA2DS2-VASc | 1.12 (0.99, 1.27) | 1.20 (1.14, 1.26) | 1.19 (1.14, 1.24) |
| **Myocardial infarction** | | | |
| Adherence (ref. non-adherence) | 0.82 (0.46, 1.45) | 0.75 (0.60, 0.94) | 0.76 (0.61, 0.94) |
| Type of OACs (ref. Warfarin) |  |  |  |
| Rivaroxaban | N/A | N/A | 0.78 (0.56, 1.10) |
| Apixaban | N/A | N/A | 0.94 (0.66, 1.35) |
| Dabigatran | N/A | N/A | 0.88 (0.60, 1.29) |
| Edoxaban | N/A | N/A | 0.63 (0.41, 0.98) |
| Sex (ref. male) | 0.63 (0.31, 1.31) | 0.54 (0.41, 0.71) | 0.55 (0.43, 0.72) |
| Age | 1.00 (0.97, 1.04) | 1.02 (1.00, 1.03) | 1.01 (1.00, 1.03) |
| CCI | 1.20 (1.06, 1.37) | 1.06 (0.99, 1.12) | 1.08 (1.02, 1.14) |
| CHA2DS2-VASc | 1.10 (0.76, 1.61) | 1.25 (1.08, 1.46) | 1.22 (1.06, 1.41) |
| **Death** | | | |
| Adherence (ref. non-adherence) | 0.55 (0.47, 0.64) | 0.54 (0.51, 0.57) | 0.54 (0.51, 0.57) |
| Type of OACs (ref. Warfarin) |  |  |  |
| Rivaroxaban | N/A | N/A | 0.69 (0.63, 0.75) |
| Apixaban | N/A | N/A | 0.79 (0.72, 0.87) |
| Dabigatran | N/A | N/A | 0.65 (0.58, 0.72) |
| Edoxaban | N/A | N/A | 0.63 (0.57, 0.70) |
| Sex (ref. male) | 0.84 (0.70, 1.00) | 0.88 (0.82, 0.95) | 0.88 (0.82, 0.94) |
| Age | 1.07 (1.06, 1.08) | 1.09 (1.09, 1.10) | 1.09 (1.08, 1.09) |
| CCI | 1.24 (1.20, 1.29) | 1.23 (1.21, 1.25) | 1.23 (1.21, 1.24) |
| CHA2DS2-VASc | 0.95 (0.86, 1.05) | 0.89 (0.85, 0.92) | 0.89 (0.86, 0.93) |
| **Intracranial hemorrhage** | | | |
| Adherence (ref. non-adherence) | 1.10 (0.73, 1.64) | 0.99 (0.82, 1.19) | 1.01 (0.85, 1.20) |
| Type of OACs (ref. Warfarin) |  |  |  |
| Rivaroxaban | N/A | N/A | 0.67 (0.53, 0.85) |
| Apixaban | N/A | N/A | 0.78 (0.60, 1.00) |
| Dabigatran | N/A | N/A | 0.41 (0.30, 0.57) |
| Edoxaban | N/A | N/A | 0.50 (0.36, 0.69) |
| Sex (ref. male) | 1.05 (0.64, 1.74) | 0.91 (0.73, 1.13) | 0.92 (0.75, 1.12) |
| Age | 1.02 (0.99, 1.04) | 1.02 (1.01, 1.03) | 1.02 (1.01, 1.03) |
| CCI | 1.13 (1.02, 1.25) | 1.06 (1.00, 1.11) | 1.06 (1.02, 1.11) |
| CHA2DS2-VASc | 1.08 (0.82, 1.42) | 0.96 (0.85, 1.09) | 0.98 (0.87, 1.10) |
| Age, CCI, and CHA2DS2-VASc are continuous variables  Abbreviations: CI, confidence interval; DOACs, direct oral anticoagulants; OACs, oral anticoagulants; CCI, Charlson comorbidity index | | | |

Supplementary Table S7. Incidence and hazard ratio by adherence

|  | **Adherent use** | | | **Non-adherent use** | | | **IRR**  **(95% CI)** | **Adjusted HR***  **(95% CI)** |
| --- | --- | --- | --- | --- | --- | --- | --- | --- |
|  | **No. of events** | **100 PY** | **IR/ 100PY** | **No. of events** | **100 PY** | **IR/ 100PY** |  |  |
| **Ischemic stroke** | | | | | | |  |  |
| Total | 2,467 | 787.5 | 3.13 | 1,441 | 340.5 | 4.23 | 0.74 (0.69, 0.79) | 0.79 (0.74, 0.85) |
| Warfarin | 197 | 56.1 | 3.51 | 241 | 59.2 | 4.07 | 0.86 (0.72, 1.04) | 0.85 (0.71, 1.03) |
| DOACs | 2,270 | 731.4 | 3.10 | 1,200 | 281.3 | 4.27 | 0.73 (0.68, 0.78) | 0.78 (0.73, 0.84) |
| Rivaroxaban | 952 | 310.5 | 3.07 | 562 | 128.3 | 4.38 | 0.70 (0.63, 0.78) | 0.76 (0.68, 0.84) |
| Apixaban | 563 | 180.0 | 3.13 | 255 | 65.5 | 3.89 | 0.80 (0.69, 0.93) | 0.87 (0.75, 1.01) |
| Dabigatran | 438 | 128.8 | 3.40 | 206 | 48.3 | 4.27 | 0.80 (0.68, 0.94) | 0.85 (0.72, 1.01) |
| Edoxaban | 317 | 112.0 | 2.83 | 177 | 39.2 | 4.52 | 0.63 (0.52, 0.75) | 0.66 (0.55, 0.79) |
| **Myocardial infarction** | | | | | | |  |  |
| Total | 234 | 806.4 | 0.29 | 140 | 349.8 | 0.40 | 0.72 (0.59, 0.89) | 0.76 (0.61, 0.94) |
| Warfarin | 21 | 57.4 | 0.37 | 27 | 60.8 | 0.44 | 0.82 (0.47, 1.46) | 0.82 (0.46, 1.45) |
| DOACs | 213 | 749.0 | 0.28 | 113 | 288.9 | 0.39 | 0.73 (0.58, 0.91) | 0.75 (0.60, 0.94) |
| Rivaroxaban | 87 | 317.8 | 0.27 | 46 | 132.2 | 0.35 | 0.79 (0.55, 1.12) | 0.80 (0.56, 1.14) |
| Apixaban | 63 | 185.0 | 0.34 | 31 | 67.1 | 0.46 | 0.74 (0.48, 1.13) | 0.78 (0.50, 1.20) |
| Dabigatran | 41 | 132.3 | 0.31 | 19 | 49.5 | 0.38 | 0.81 (0.47, 1.39) | 0.86 (0.50, 1.49) |
| Edoxaban | 22 | 113.9 | 0.19 | 17 | 40.2 | 0.42 | 0.46 (0.24, 0.86) | 0.46 (0.24, 0.86) |
| **Death** | | | | | | |  |  |
| Total | 2,726 | 808.9 | 3.37 | 2,583 | 350.8 | 7.36 | 0.46 (0.43, 0.48) | 0.54 (0.51, 0.57) |
| Warfarin | 257 | 57.6 | 4.46 | 494 | 61.0 | 8.10 | 0.55 (0.47, 0.64) | 0.55 (0.47, 0.64) |
| DOACs | 2,469 | 751.2 | 3.29 | 2,089 | 289.8 | 7.21 | 0.46 (0.43, 0.48) | 0.54 (0.51, 0.57) |
| Rivaroxaban | 1,000 | 318.8 | 3.14 | 875 | 132.6 | 6.60 | 0.48 (0.43, 0.52) | 0.57 (0.52, 0.62) |
| Apixaban | 746 | 185.6 | 4.02 | 638 | 67.3 | 9.48 | 0.42 (0.38, 0.47) | 0.51 (0.46, 0.57) |
| Dabigatran | 377 | 132.8 | 2.84 | 292 | 49.7 | 5.87 | 0.48 (0.41, 0.56) | 0.56 (0.48, 0.65) |
| Edoxaban | 346 | 114.1 | 3.03 | 284 | 40.2 | 7.06 | 0.43 (0.37, 0.50) | 0.49 (0.42, 0.58) |
| **Intracranial hemorrhage** | | | | | | |  |  |
| Total | 438 | 805.3 | 0.54 | 205 | 349.3 | 0.59 | 0.93 (0.79, 1.09) | 1.01 (0.85, 1.20) |
| Warfarin | 49 | 57.3 | 0.86 | 47 | 60.6 | 0.78 | 1.10 (0.74, 1.65) | 1.10 (0.73, 1.64) |
| DOACs | 389 | 748.1 | 0.52 | 158 | 288.6 | 0.55 | 0.95 (0.79, 1.14) | 0.99 (0.82, 1.19) |
| Rivaroxaban | 180 | 317.3 | 0.57 | 69 | 132.1 | 0.52 | 1.09 (0.82, 1.43) | 1.14 (0.86, 1.50) |
| Apixaban | 117 | 184.5 | 0.63 | 52 | 66.8 | 0.78 | 0.82 (0.59, 1.13) | 0.83 (0.60, 1.16) |
| Dabigatran | 42 | 132.5 | 0.32 | 20 | 49.6 | 0.40 | 0.79 (0.46, 1.34) | 0.80 (0.47, 1.38) |
| Edoxaban | 50 | 113.8 | 0.44 | 17 | 40.1 | 0.42 | 1.04 (0.60, 1.80) | 1.08 (0.62, 1.87) |
| *The hazard ratio was adjusted by age, sex, the Charlson comorbidity index, and the CHA2DS2-VASc score.  Abbreviations: PY, person-years; IR: incidence rate; IRR, incidence rate ratio; CI, confidence interval; DOACs, direct oral anticoagulants | | | | | | | | |

**Supplementary Table S8.** Hazard ratios of oral anticoagulants

|  | **Hazard ratio (95% CI)** | | | | |
| --- | --- | --- | --- | --- | --- |
|  | **Warfarin** | **Rivaroxaban** | **Apixaban** | **Dabigatran** | **Edoxaban** |
| **Ischemic stroke** |  |  |  |  |  |
| Total | 1.00 | 0.92 (0.83, 1.03) | 0.82 (0.73, 0.93) | 1.00 (0.88, 1.13) | 0.81 (0.71, 0.92) |
| Age<75 | 1.00 | 0.85 (0.72, 1.00) | 0.88 (0.73, 1.06) | 0.93 (0.77, 1.13) | 0.80 (0.65, 0.99) |
| Age≥75 | 1.00 | 0.98 (0.85, 1.13) | 0.81 (0.69, 0.95) | 1.05 (0.89, 1.24) | 0.82 (0.69, 0.97) |
| CHA2DS2-VASc<4 | 1.00 | 0.93 (0.81, 1.06) | 0.82 (0.71, 0.96) | 0.99 (0.85, 1.15) | 0.78 (0.66, 0.92) |
| CHA2DS2-VASc≥4 | 1.00 | 0.91 (0.76, 1.10) | 0.83 (0.68, 1.01) | 1.01 (0.82, 1.25) | 0.86 (0.69, 1.07) |
| CCI<3 | 1.00 | 0.99 (0.85, 1.16) | 0.82 (0.69, 0.98) | 1.02 (0.85, 1.22) | 0.89 (0.74, 1.07) |
| CCI≥3 | 1.00 | 0.86 (0.74, 1.00) | 0.83 (0.71, 0.98) | 0.99 (0.83, 1.17) | 0.73 (0.60, 0.88) |
| **Myocardial infarction** |  |  |  |  |  |
| Total | 1.00 | 0.78 (0.56, 1.10) | 0.94 (0.66, 1.35) | 0.88 (0.60, 1.29) | 0.63 (0.41, 0.98) |
| Age<75 | 1.00 | 0.86 (0.54, 1.38) | 0.98 (0.58, 1.64) | 0.65 (0.36, 1.17) | 0.67 (0.35, 1.29) |
| Age≥75 | 1.00 | 0.74 (0.46, 1.20) | 0.94 (0.57, 1.54) | 1.08 (0.64, 1.82) | 0.62 (0.34, 1.10) |
| CHA2DS2-VASc<4 | 1.00 | 0.85 (0.56, 1.30) | 1.03 (0.66, 1.62) | 0.91 (0.56, 1.48) | 0.81 (0.49, 1.36) |
| CHA2DS2-VASc≥4 | 1.00 | 0.67 (0.38, 1.16) | 0.80 (0.45, 1.43) | 0.82 (0.43, 1.54) | 0.34 (0.15, 0.79) |
| CCI<3 | 1.00 | 1.42 (0.81, 2.46) | 1.43 (0.79, 2.59) | 0.91 (0.47, 1.77) | 1.12 (0.58, 2.14) |
| CCI≥3 | 1.00 | 0.46 (0.29, 0.73) | 0.72 (0.46, 1.14) | 0.91 (0.57, 1.46) | 0.39 (0.21, 0.73) |
| **Death** |  |  |  |  |  |
| Total | 1.00 | 0.69 (0.63, 0.75) | 0.79 (0.72, 0.87) | 0.65 (0.58, 0.72) | 0.63 (0.57, 0.70) |
| Age<75 | 1.00 | 0.65 (0.56, 0.76) | 0.77 (0.65, 0.92) | 0.51 (0.41, 0.63) | 0.60 (0.48, 0.75) |
| Age≥75 | 1.00 | 0.73 (0.66, 0.81) | 0.83 (0.74, 0.92) | 0.72 (0.64, 0.81) | 0.66 (0.59, 0.75) |
| CHA2DS2-VASc<4 | 1.00 | 0.67 (0.61, 0.75) | 0.80 (0.72, 0.9) | 0.63 (0.55, 0.71) | 0.65 (0.57, 0.73) |
| CHA2DS2-VASc≥4 | 1.00 | 0.73 (0.62, 0.85) | 0.77 (0.66, 0.91) | 0.69 (0.58, 0.83) | 0.61 (0.5, 0.74) |
| CCI<3 | 1.00 | 0.79 (0.69, 0.90) | 0.95 (0.82, 1.09) | 0.77 (0.66, 0.91) | 0.78 (0.66, 0.92) |
| CCI≥3 | 1.00 | 0.65 (0.58, 0.73) | 0.72 (0.64, 0.81) | 0.58 (0.51, 0.67) | 0.55 (0.48, 0.64) |
| **Intracranial hemorrhage** |  |  |  |  |  |
| Total | 1.00 | 0.67 (0.53, 0.85) | 0.78 (0.60, 1.00) | 0.41 (0.30, 0.57) | 0.50 (0.36, 0.69) |
| Age<75 | 1.00 | 0.60 (0.42, 0.84) | 0.84 (0.58, 1.22) | 0.39 (0.25, 0.63) | 0.46 (0.28, 0.75) |
| Age≥75 | 1.00 | 0.74 (0.53, 1.04) | 0.76 (0.53, 1.10) | 0.44 (0.28, 0.69) | 0.54 (0.36, 0.83) |
| CHA2DS2-VASc<4 | 1.00 | 0.79 (0.59, 1.06) | 0.81 (0.59, 1.11) | 0.42 (0.28, 0.62) | 0.53 (0.36, 0.78) |
| CHA2DS2-VASc≥4 | 1.00 | 0.44 (0.29, 0.67) | 0.70 (0.46, 1.08) | 0.41 (0.24, 0.71) | 0.45 (0.26, 0.78) |
| CCI<3 | 1.00 | 0.90 (0.63, 1.27) | 0.93 (0.64, 1.36) | 0.43 (0.27, 0.68) | 0.65 (0.42, 1.00) |
| CCI≥3 | 1.00 | 0.48 (0.34, 0.68) | 0.67 (0.47, 0.95) | 0.43 (0.28, 0.67) | 0.38 (0.24, 0.62) |

Supplementary Figure S1. Cohort study design for identifying the correlation between early and later adherence


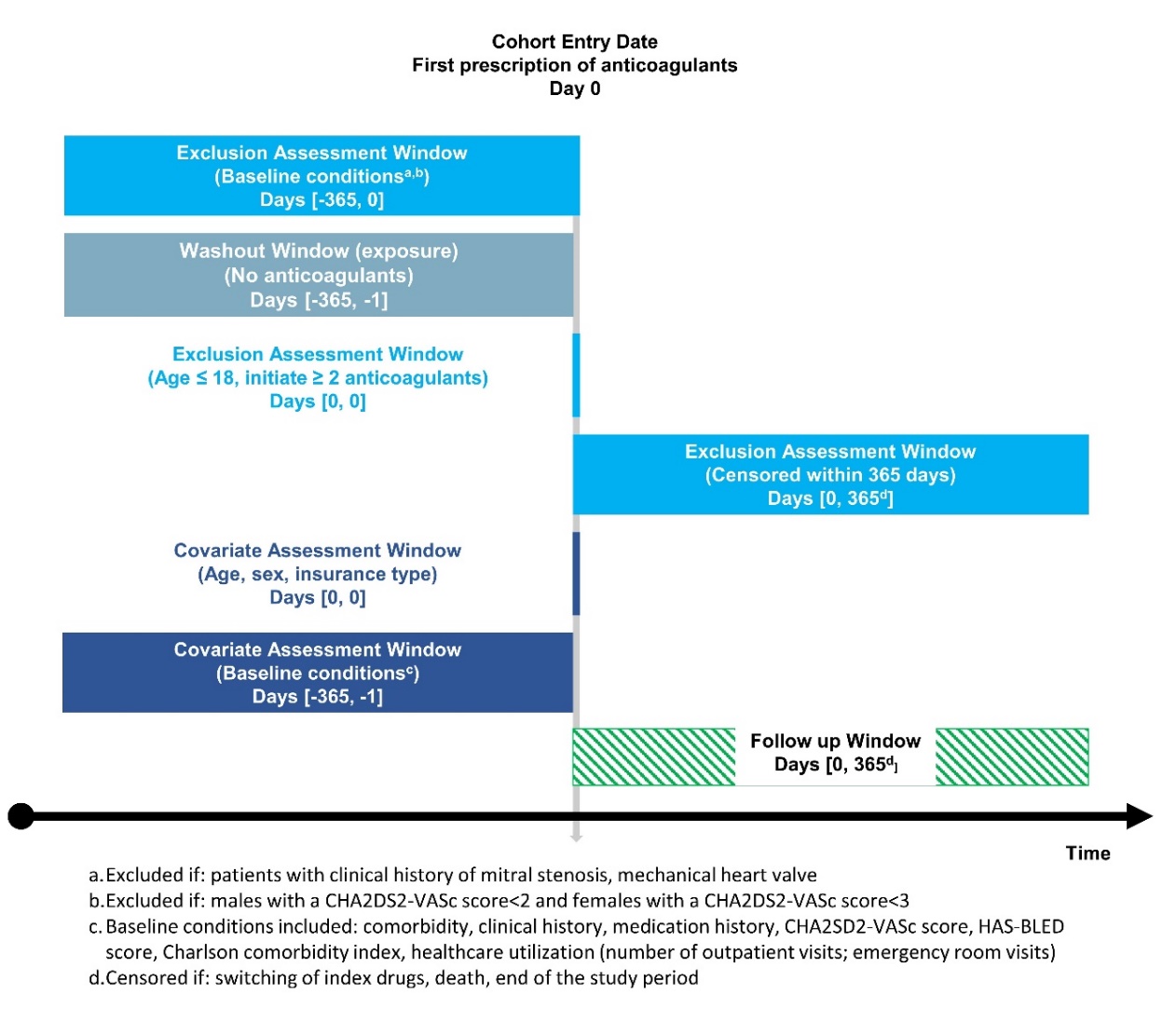


Supplementary Figure S2. Selection of study population for identifying the association between early and later adherence


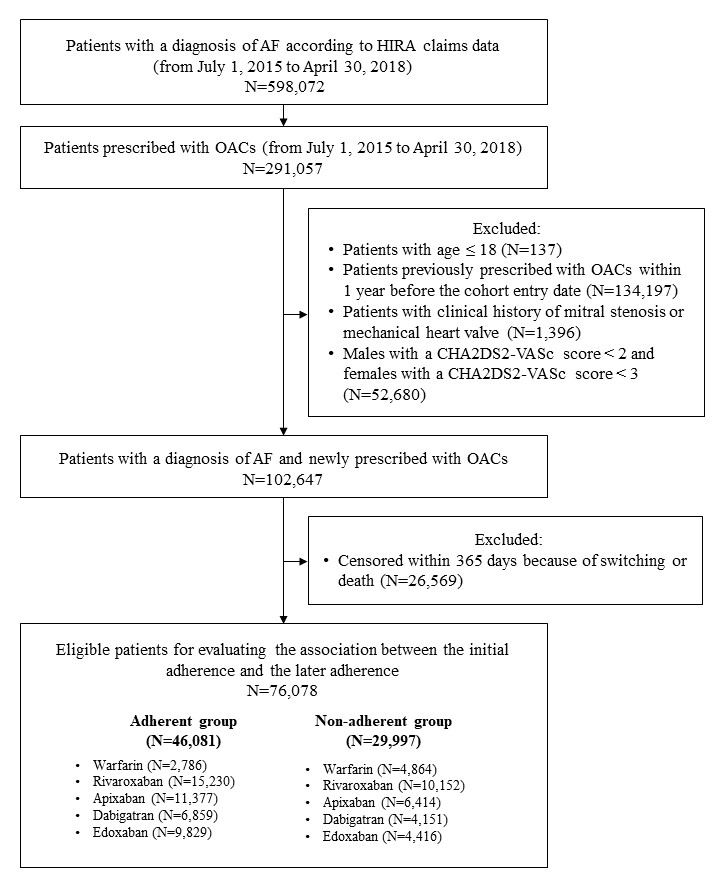


AF, atrial fibrillation; HIRA, Health Insurance Review and Assessment Service; OACs, oral anticoagulants.

Supplementary Figure S3. Scatter plot between early and later oral anticoagulant adherence


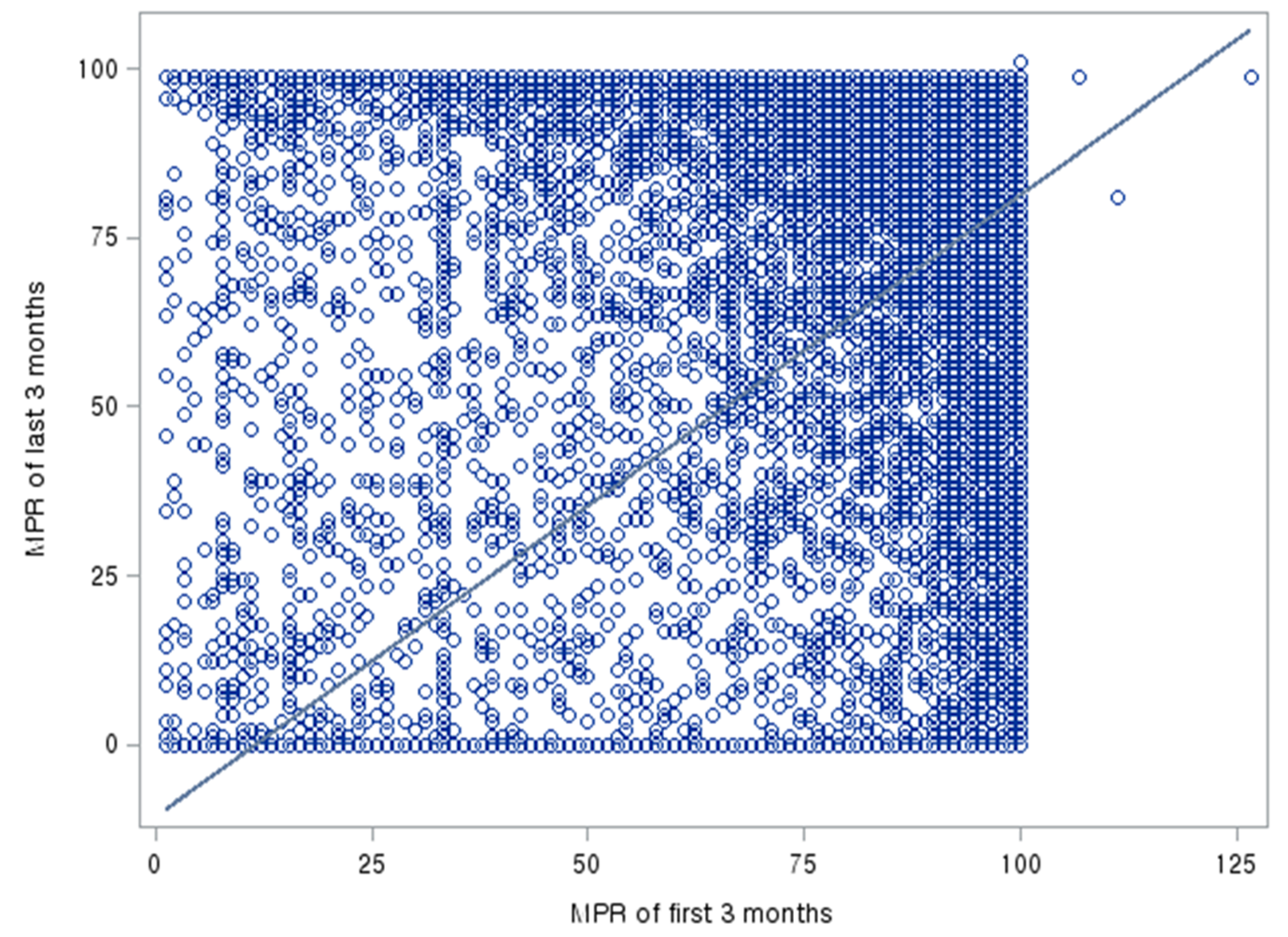


Blue line: regression line

MPR, medication possession rate.
